# Development of scales to assess children's perceptions of friend and parental influences on physical activity

**DOI:** 10.1186/1479-5868-6-67

**Published:** 2009-10-12

**Authors:** Russell Jago, Kenneth R Fox, Angie S Page, Rowan Brockman, Janice L Thompson

**Affiliations:** 1 Department of Exercise, Nutrition & Health Sciences, University of Bristol, Bristol, UK

## Abstract

**Background:**

Many children do not meet physical activity guidelines. Parents and friends are likely to influence children's physical activity but there is a shortage of measures that are able to capture these influences.

**Methods:**

A new questionnaire with the following three scales was developed: 1) *Parental influence on physical activity*; 2) *Motives for activity with friends *scale; and 3) *Physical activity and sedentary group normative values*. Content for each scale was informed by qualitative work. One hundred and seventy three, 10-11 year old children completed the new questionnaire twice, one week apart. Participants also wore an accelerometer for 5 days and mean minutes of moderate to vigorous physical activity, light physical activity and sedentary time per day were obtained. Test-retest reliability of the items was calculated and Principal Component analysis of the scales performed and sub-scales produced. Alphas were calculated for main scales and sub-scales. Correlations were calculated among sub-scales. Correlations between each sub-scale and accelerometer physical activity variables were calculated for all participants and stratified by sex.

**Results:**

The Parental influence scale yielded four factors which accounted for 67.5% of the variance in the items and had good (α > 0.7) internal consistency. The Motives for physical activity scale yielded four factors that accounted for 66.1% and had good internal consistency. The Physical activity norms scale yielded 4 factors that accounted for 67.4% of the variance, with good internal consistency for the sub-scales and alpha of .642 for the overall scale. Associations between the sub-scales and physical activity differed by sex. Although only 6 of the 11 sub-scales were significantly correlated with physical activity there were a number of associations that were positively correlated >0.15 indicating that these factors may contribute to the explanation of children's physical activity.

**Conclusion:**

Three scales that assess how parents, friends and group normative values may be associated with children's physical activity have been shown to be reliable and internally consistent. Examination of the extent to which these new scales improve our understanding of children's physical activity in datasets with a range of participant and family characteristics is needed.

## Background

Regular physical activity has many short and long-term benefits for children including lower body mass index [1] and lower mean values for cardiovascular risk factors [2-4]. Physical activity is also associated with higher levels of mental well-being among children [5] and helps children to develop social skills [6,7]. Despite these benefits many children and adolescents do not engage in recommended amounts of physical activity [8,9]. The mediating variable model suggests that in order to change a behavior (such as physical activity) we need to change the key factors or mediators of that behavior [10]. Therefore, in order to develop effective interventions to increase children's physical activity we need to understand the factors, or correlates of children's physical activity and then change those variables [11].

Parents and friendship groups are likely to be key influences on children's physical activity behaviors. Davison and colleagues reported that fathers' modeling of active behaviors and mothers' logistic support for physical activity (e.g., enrolling child in sport programs and going to sporting events with the child) were associated with the physical activity levels of 9 year old girls [12]. Salvy and colleagues reported that 10 year old boys and girls were more likely to engage in high intensity physical activity with friends [13] and that friends increased children's motivation for physical activity. Peer support for physical activity has also indicated an association with higher amounts of physical activity among fifth to eighth grade US students [14]. While these studies demonstrate the importance of the influence of parents and friends on children's physical activity they provide limited information about how these influences are manifested. More information about potential mechanisms through which interactions with parents and friends shape physical activity behaviors is therefore needed to develop effective strategies to increase children's physical activity.

Our research team have conducted extensive qualitative work to examine how friends and parents influence the physical activity behaviors of 10-11 year old children [15-17]. The data on the influence of friends showed that 10-11 year old British children have three types of friendship groups: school friends, neighborhood friends and other friends (e.g. children of their parents' friends and children from youth or community groups) with most children belonging to multiple groups [15]. Findings also suggested that friendship group members shared common attitudes and perceived normative values for physical activity and screen-viewing [15]. Children also reported that their reasons or motives for participating in physical activity included intrinsic appeal, increasing social affiliation and preventing isolation [15]. Motives for physical activity participation were also influenced by group affiliation [15]. Therefore, understanding group affiliation may be important for understanding physical activity attitudes, expectations and norms among children.

Our qualitative work indicated that parents exert considerable influence on children's behavior through provision of transport assistance, financial support, modeling, encouragement, and setting rules for activity [16,17]. Several participants reported that their family structure and particularly the parents or guardians who they live with differ for weekday and weekend days. This suggests that measures should accommodate these differences when examining parental influence.

There is a need to understand how friends and parental factors influence children's physical activity, parental rules for physical activity, and physical activity and screen-viewing subjective normative values. Subjective normative values are a key component of the Theory of Planned Behavior and represent a person's perception of other peoples (i.e. other children's) preferences for engaging in a behavior such as physical activity [18]. However, although there are reliable measures that capture parental support for physical activity [12] there are no scales that also examine parent imposed activity-related rules, or scales that can address how associations may differ by weekday or weekend parent. Similarly, while some measures have included items that address the extent to which spending time with friends contribute to physical activity enjoyment [19] to our knowledge no current measure identifies friend influences on physical activity and particularly if influence differs by type of friend. Finally, although physical activity norms and motives for physical activity scales have been developed these scales have tended to focus on more global subjective norms and included questions on fellow students, teacher and parent norms [20] and have not included screen-viewing behaviors. To address these limitations this paper reports the development and reliability assessment of three new questionnaire scales to assess parental influence on physical activity, motives for activity with friends, and group norms related to physical activity and sedentary behaviors.

## Methods

Participants were 173, 10-11 year old children recruited from 7 primary schools in Bristol, England. We initially approached 9 primary schools with 2 schools declining, one because of recent changes to the school management team and the other due to participation in a number of other, non-physical activity based research projects. There were 373, Year 6 pupils within the 7 primary schools and as such the recruitment rate was 46.4%. Participant sex and highest education within the household were obtained by parental report. The study was approved by a University of Bristol ethics committee and informed consent and assent were obtained for all participants [21].

### Questionnaire development

The questionnaire included three main scales: 1) *Parental influence on physical activity *scale which included questions about the parents that children spent time with on both weekdays and weekend days; 2) *Motives for activity with friends *scale which examined reasons for participating in physical activity and whether motives were different for school friends, neighborhood friends or other friends; and 3) *Physical activity and sedentary group normative *(norms) values. Content for each scale was derived from our qualitative work with these populations [15-17]. For example, a number of the children who took part in focus groups reported that social factors, prevention of bullying and isolation and spending time with friends influenced their participation in physical activity with participants also expressing diverse views about the merits and perceived negative connotations of participation in team sports, and screen-viewing behaviors. Items that were designed to capture all of these issues were generated by the first author and then reviewed, modified and added to by all of the co-authors. This process was repeated several times until all of the study team were satisfied with the items. Items were phrased as statements to which participants were provided with four response options: disagree a lot; disagree; agree; and agree a lot. The response options were modeled after the responses for an Australian survey [22] which included two additional options for neither (disagree or agree) or don't know. These two options were omitted from the survey as our pilot work indicated that UK children found these two options confusing.

### Procedures

Height was measured to the nearest mm with a SECA Leicester Stadiometer. Weight was measured to the nearest 0.1 kg using a SECA 899 digital scale. Body mass index (BMI) was calculated (kg/m^2^). Participants completed the new questionnaire twice, with the second administration approximately a week after the first. Physical activity was objectively measured using an accelerometer (Actigraph GT1M; Actigraph LLC USA) programmed to record data every 10 seconds. Children wore the accelerometer on a belt around their waist during waking hours for five consecutive days, including weekends. At the end of the measurement period the monitors were collected by researchers and the data downloaded to a PC. Any 20 minute periods of zero activity recorded by the accelerometer were taken to indicate that the accelerometer was not being worn and were classified as missing data. Participants were included in the analysis if they provided at least 500 minutes of data for at least 3 days. It has previously been reported that between 3 and 7 days of accelerometer data are required to provide an indication of habitual physical activity using accelerometers [23-25]. Therefore, we employed a three day inclusion criteria as the minimum threshold for our data. While we accept that it has been suggested that more than 3 days might be needed to capture the less predictable behavior of children [23,25], the 3-day inclusion criteria has been widely used for children and adolescents [26-28] and provided us with the largest possible sample size. Mean counts per minute were calculated to provide an indication of the overall volume of physical activity in which the participants engaged. Mean minutes of sedentary, light and moderate to vigorous physical activity per day were also calculated using child-specific cut-points [29]. However, as the count values derived from the GT1M are 9% higher than those obtained from the original 7164 monitors which were used to derive intensity thresholds, a correction factor of 0.91 was used for all intensity values [30].

### Statistical analyses

Descriptive statistics including means, standard deviations and percents were calculated for all demographic and physical activity variables. Initial checks indicated that there was variance in responses to each item. As the aim of this paper was to develop questionnaires with good test-retest reliability, paired t-tests were used to assess the test-retest reliability of individual items prior to factor analysis. Items that were significantly different (P < .001) between the two administrations were omitted from further analysis. Pearson correlations were then conducted between the first and second administrations of the items and any items that were not associated were not included in further analysis.

For items that were retained after the initial reliability analyses we also calculated test retest intra-class correlations (ICC). Although intra-class correlations have been frequently recommended for reliability studies [31-33] authors have commented on the number of different ICC's that could be used for reliability studies and the lack of a clear consensus on when and why to select a particular type of ICC [31,33]. We performed two-way random effect (subjects and time are both random) intra-class correlations that assessed absolute agreement (as ideally you would want the same response two weeks apart). There is also a debate about the criteria that should be used to assess the reliability ICC's. A number of authors [34,35] have applied the "benchmark" criteria of Landis and Koch that was initially described for Kappa statistics [36]. According to these criteria test re-test ICC's are interpreted as: 0.21 - 0.40 Fair agreement, 0.41 - 0.60 Moderate agreement, 0.61 - 0.80 Substantial agreement and 0.81 - 1.00 Almost perfect agreement [36]. In light of the uncertainty over how to apply these criteria we opted to remove any item that had an ICC that was less than 0.4. However, in light of the ambiguity on how to apply and interpret the ICC's we report the ICC's for each item retained in the analysis but did not apply any further exclusion criteria. In terms of interpretation ICC's of 0.4 -0.59 were considered acceptable but improvable, 0.60- 0.79 satisfactory, ≥ 0.80 excellent [35].

Once reliable items had been identified Principal Component analysis with Varimax rotation was then conducted separately for each of the three scales. The resulting scree plots and eigen-values were inspected, interpretability considered, and factors selected. Items that did not load on factors (at least 0.4), or loaded on multiple factors were removed and the models re-run. Values for items included in rotated factors were summed and used in analyses. The internal consistency of all of the items included in each resulting factor was then assessed using Cronbach's Alpha. Alpha was then also calculated for all of the items that were retained in each of the three overall scales.

Bivariate Pearson correlations were used to examine inter-relationships between each of the factor scores that were derived from the Principal Component analysis. To provide an indication of the relevance of these measures in relation to physical activity bivariate Pearson correlations were then conducted between the factor scores and all four of the accelerometer derived physical activity variables. However, since extensive research has shown that children's physical activity [8] differ by sex and that the associations between psychosocial variables and children's physical activity differ by sex [37,38], correlations with physical activity were calculated for all participants and then stratified by sex.

## Results

Demographic characteristics for the 173 participants in the study are shown in Table 1. The sample was 51% female with 47% living in households in which the highest level of education was GCSE (national school examinations assessed at age 16). Valid accelerometer data were obtained for 131 participants with the participants obtaining an average of 21.5 minutes of moderate to vigorous intensity physical activity per day.

**Table 1:** Participant characteristics

	**n**	**%**
Male	86	49.1
Female	89	50.9
		
Highest level of Education		
GCSE or similar	83	47.4
A'Level or similar	33	18.9
Degree	36	21.7
Higher degree	13	7.4
Missing	8	4.6
		
	**n**	**Mean (SD)**

BMI	173	19.0 (6.6)
Accelerometer minutes of Moderate to Vigorous Physical Activity per day	131	21.5 (12.3)
Accelerometer minutes of Light Activity per day	131	121.2 (25.6)
Accelerometer minutes of sedentary time per day	131	1217.3 (56.8)
Accelerometer counts per minute	131	457.7 (111.6)

The final *Parental influence on physical activity *scale is presented in Table 2. No items were dropped from the scale which included 14 items and accounted for 67.5% of the overall variance and had a reasonable internal consistency (alpha = .75). The

**Table 2:** Parental influence on children's physical activity scale and factor structure

	**General parenting support**	**Active parents**	**Past activity**	**Guiding support**
PERCENT VARIANCE EXPLAINED	23.2%	19.4%	12.5%	12.4%

ALPHA FOR SUB-SCALE	.830	.836	.802	.819

The adult(s) I live with on a weekend day pay for me to take part in physical activity (for example paying for swimming or to attend football club)	**.833**	.041	.069	.038
The adult(s) I live with on a weekend day drive me to sports clubs	**.774**	-.056	.071	.133
The adult(s) I live with on a weekday pay for me to take part in physical activity (for example paying for swimming or to attend football club)	**.760**	.047	.027	-.115
The adult(s) I live with on a weekday take me to or collect me from sport or exercise clubs	**.759**	.085	-.177	.059
The adult(s) I live with on a weekend day encourage (or tell) me to be physically active	**.632**	.204	.017	-.006
The adult(s) I live with on a weekday encourage (or tell) me to be physically active	**.577**	.284	.074	-.044
				
The adult(s) I live with on a weekend day take part in lots of physical activity	.172	**.812**	-.216	.024
The adults(s) I live with on a weekend day take part in physical activity with me	.044	**.810**	.082	.002
The adult(s) I live with on a weekday take part in physical activity with me	.072	**.787**	.086	.119
The adult(s) I live with on a weekday take part in lots of physical activity	.145	**.786**	-.090	.037
				
The adult(s) I live with on a weekday used to take part in lots of physical activity but they don't anymore	.005	-.015	**.906**	.031
The adult(s) I live with on a weekend day used to take part in lots of physical activity but they don't anymore	.069	-.056	**.888**	.209
				
The adult(s) I live with on a weekday have rules for physical activity (such as being home at a set time, not going to some places etc)	-.007	.036	.099	**.908**
The adult(s) I live with on a weekend day have rules for physical activity (such as being home at a set time, not going to some places etc)	.038	.113	.120	**.904**

**Alpha for overall Scale = .746**	**Overall variance explained = 67.5%**			

*The General parenting support *scale provides an indication of the overall support that the child perceives their parent provides for physical activity (23.2% of the variance, alpha = .83) while the *Active parents *sub-scale provides a measure of the extent to which the child perceives his or her parent to be active (19.4% of the variance, alpha = .84). *Past parental activity *provides an indication whether or not the child perceives that the parental used to be active (12.5% of the variance, alpha = .80) while the *Guiding support *(12.5% of the variance, alpha = .82) scale captures the extent to which the child's parents have supportive rules for physical activity participation. Intra-class correlations for all items included in the Parental influence on physical activity were between 0.6 and 0.8 (7 > 0.7) suggesting satisfactory reliability.

The final *Motives for physical activity with friends *scale is presented in Table 3. Three items were not included in the factor analysis due to poor reliability, with an additional four items removed from the scale because they cross-loaded onto multiple factors. The scale had 14 items that accounted for 66.1% of the overall variance and had good internal consistency (alpha = 0.86). The *Prevent bullying *sub-scale identifies the extent to which the child is motivated to engage in activity to prevent peer victimization (18.6% of the overall variance, alpha = .79). The *Social sedentary *sub-scale identifies the extent to which a child engages in sedentary behaviours for social reasons (16.8% of the variance, alpha = .76) while the *Social affiliation *sub-scale (15.6% of the variance alpha = .71) identifies the extent to which group affiliation influences activity participation. Finally, the *Neighborhood friends *sub-scale (15.2% of the variance, alpha = .79) identifies the extent to which children are specifically motivated to engage in physical activity to spend time with children who live in their neighborhood. The intra-class correlation for the first item on the Prevent Bullying sub-scale was excellent (.845), 10 further items had satisfactory ICC's (7 > 0.7) while the remaining 3 items had ICC's that were considered acceptable but improvable ['I take part in sitting down activity to spend time with my friends' (.522); 'I take part in physical activity because my friends at school do' (.504); and 'I take part in physical activity because my neighborhood friends do' (.564)].

**Table 3:** Motives for activity with friends' scale and factor structure

	**Prevent bullying**	**Social Sedentary**	**Social affiliation**	**Neighborhood friends**
PERCENT VARIANCE EXPLAINED	18.6%	16.8%	15.6%	15.2%

ALPHA FOR SUB-SCALE	.789	.761	.714	.793

I take part in physical activity with my other friends so that I don't get picked on	**.822**	.075	.254	.056
I take part in physical activity with my neighborhood friends so that I don't get picked on	**.783**	.117	.018	.264
I take part in physical activity at school so that I don't get picked on	**.745**	.112	.195	.171
I take part in sitting down activity because my other friends do	**.510**	.391	.216	.051
				
I take part in sitting down activity to spend time with my other friends	.108	**.844**	.102	.093
I take part in sitting down activity to spend time with my school friends	.009	**.814**	.138	.061
I take part in sitting down activity because I want to belong in a group with my other friends	.318	**.684**	.153	.213
				
I take part in physical activity because my friends at school do	.183	.076	**.762**	-.046
I take part in physical activity to spend time with my friends	-.027	.204	**.759**	.155
I take part in physical activity because my other friends do	.363	.017	**.653**	.099
I take part in physical activity because I want to belong in a group with my other friends	.284	.335	**.519**	.200
				
I take part in physical activity to spend time with my neighborhood friends	.077	.061	.132	**.917**
I take part in sitting down activity to spend time with my neighborhood friends	.178	.395	-.118	**.740**
I take part in physical activity because my neighborhood friends do	.321	.029	.302	**.703**

**Alpha for overall Scale = .857**		**Overall variance explained = 66.1%**		

One item was removed from the final *Physical activity and sedentary norms scale *because the test retest ICC was low (.334) but all other original items were retained in a scale that accounted for 67.9% of the overall variance and had an alpha of .64 (Table 4). The *Sedentary *sub-scale identifies norms for screen-viewing behaviors (28.9% of the variance) with an *Activity *sub-scale providing comparable information for physical activity norms (17.6% of the variance). The analysis also yielded a *Teasing *sub-scale (20.8% of the variance) which provides information on the extent to which respondents feel that they would be teased for engaging in physical activity and screen-viewing. The ICC's for the retained variables indicated that 8 items had satisfactory reliability (>0.6) and 2 items [Kids my age spend lots of time watching TV or DVD's = .44, Kids my age think taking part in physical activity is a good thing to do = .55] had acceptable but improvable reliability.

**Table 4:** Physical activity norms scale and factor structure

	**Sedentary**	**Teasing**	**Activity**
PERCENT VARIANCE EXPLAINED	28.9%	20.8%	17.6%
ALPHA FOR SUB-SCALE	.795	.709	.701

Kids my age think watching TV/DVDs is a good thing to do	**.829**	-.014	-.260
Kids my age think playing computer games (such as XBOX, PlayStation or Nintendo) is a good thing to do	**.830**	-.062	-.170
Kids my age spend lots of time playing on games consoles (such as XBOX, PlayStation or Nintendo)	**.765**	.133	.275
Kids my age spend lots of time watching TV or DVD's	**.710**	.238	.213
			
Kids my age would tease me if I spent a lot of time taking part in physical activity	.179	**.870**	-.107
Kids my age would tease me if I went to lots of after-school sport or other sports clubs	.129	**.859**	-.142
Kids my age would tease me if I spend a lot of time playing computer games (such as XBOX, PlayStation or Nintendo)	-.075	**.734**	.066
			
Kids my age think attending after-school or sports clubs is a good thing	.012	-.124	**.783**
Kids my age think taking part in physical activity is a good thing to do	-.132	-.178	**.807**
Kids my age take part in lots of physical activity	.104	.123	**.701**

**Alpha for overall Scale = .642**	**Overall variance = 67.4%**		

The associations among the 11 sub-scales are presented in Table S1 (Additional File 1). There were a number of statistically significant associations among the sub-scales with the strongest associations within the same scale being the *Avoid bullying *and *Social affiliation *sub-scales of the motives for activity with friends scale (r = .521, p < .001). There were also significant associations between sub-scales derived from different main scales for example *Teasing norms *was significantly associated with the *Avoid bullying *sub-scale of the motives for activity overall scale (r = .427, p < .001).

Correlation analyses indicated that when the sample included all participants, the *Active parent *sub-scale of the parental influence scale was associated with light intensity physical activity (r = .178, p = .042) while *Active norms *was associated with minutes of MVPA per day (r = .181, p = .039) (data not in tabular form). Pearson correlations between each of the 11 sub-scales and the four physical activity variables are presented separately for boys and girls in Table S2 (Additional File 2). Among girls, *Social sedentary *was associated with sedentary minutes per day (r = .273, p = .024) and negatively associated with mean counts per minute (r = -.249, p = .040). *Parental past activity *was negatively associated with minutes of light activity per day (r = -.307, p = .011) and *Avoid bullying *was negatively associated with minutes of MVPA per day (r = -.255, p = .037) and accelerometer counts per minute (r = -.268, p = .028). For boys, *Social affiliation *was associated with minutes of MVPA per day (r = .253, p = .050).

## Discussion

In this paper we have presented information on the factor structure and reliability of three new scales: the *Parental influence on physical activity, Motives for activity with friends *and *Physical activity and sedentary norms*. Items were only included in the scales if they had acceptable test - retest reliability and variance in responses and thus all scales can be considered reliable. The alphas for the *Parental influence on physical activity *and *Motives for activity with friends scales *as well as the alphas for all of the sub-scales of these measures were >0.7. Alpha values >0.7 are considered satisfactory for non-clinical instruments [39] and therefore we can be confident that the items included in these scales were measuring coherent concepts. The alpha for the overall *Norms *scale was .64 indicating that caution is required when attempting to use all of the items on this overall scale to describe friend related physical activity norms. Collectively, these analyses therefore highlight that we have developed new scales that have good test re-test reliability and internal consistency.

The associations between the sub-scales and physical activity were different for boys and girls, suggesting that the extent to which new sub-scales predicted physical activity differed by sex. For example, the correlation between *Parental past activity *and light intensity physical activity was -.307 for girls but there was no association for boys. This would suggest that parental physical activity influences girl's physical activity only. Similarly, the *Social sedentary *scale correlated .275 with minutes of sedentary time for girls but there was no association for boys. These findings are consistent with previous research which has shown that the association between correlates of children's physical activity differs by gender. For example, gender differences in the associations between self-efficacy, social norms, beliefs, and outcomes of children and adolescents have been reported [37,40]. Findings suggest that the parental and friendship factors derived from our new questionnaire could be important predictors of behavior, but associations may well be sex specific and thus further research that examines these differences is required.

Although not statistically significant (p < .05), a number of the sex stratified associations between the sub-scales and physical activity variables were in excess of 0.15 and often above 0.20. Such associations are comparable to the associations between physical activity self-efficacy and physical activity [40-42]. A number of physical activity interventions have been designed in which self-efficacy is hypothesized to be a key mediator of physical activity behavior change [43,44]. Although there is a shortage of studies that have employed mediating variable analyses of self-efficacy based interventions [11,45] this is likely to be a function of the lack of success in changing youth physical activity which hampers the detection of mediation effects [45]. The comparison is salient because it suggests that these sub-scales could explain a considerable amount of the variance in children's physical activity behaviors. Therefore an appropriately powered study is needed to fully examine the associations between these constructs and children's physical activity.

The *Prevent bullying *sub-scale accounted for the highest amount of variance (18.6%) of the four factors on *Motives for activity with friends *scale, suggesting that this concept is particularly salient for some participants. Interestingly, *Teasing *was a specific sub-scale on the *Norms *scale suggesting that this factor was also a salient normative value. Being physically active is associated with a reduction in the likelihood that 13-15 year old adolescents are bullied [46]. Furthermore, athletic identity is associated with increased physical activity among children [47] and many children, particularly girls, are socialized out of physical activity by teasing or peer victimization on the basis of poor sporting ability [48]. As such these two sub-scales may identify children who are concerned about or perhaps at risk of peer teasing. The scales could also be utilized as a means of identifying particular groups of children who do not engage in key behaviors because of fears of social isolation and teasing.

The *General parenting support *and *Active parents *sub-scales include similar items to Davison's logistic support factor [12] but utilize more specific examples and have resulted in two factors rather than one. An interesting area of future research would therefore be to consider how the new sub-scales and Davison's measure are related to each other and whether utilizing all scales increases our understanding of how parents can help to support physical activity.

The *Neighborhood friends *sub-scale suggests that there is something specific about how children identify with this group of friends. As the concept of neighborhood friends and their influence on physical activity behaviors is new [15], exploring the role of this friendship group and how neighborhood friends can help promote activity in less active children is likely to be essential in fully understanding why different groups of children are active.

In the *Motives for Activity with Friends *scale there were two social sub-scales: *Social sedentary *which captured preferences for engaging in sedentary behaviors with friends; and *Social affiliation *which captured engaging in activity to spend time with friends. A number of studies have reported that social factors are associated with participation in physical activity [15,49-51], and these scales extend that work by indicating that the social aspects of screen-viewing and physical activity are likely to be different. The increased specificity of these sub-scales suggest that they may be able to explain more of the variance in the behaviors to which they relate than current measures and research that focuses on this possibility is needed. Moreover, like the teasing and prevent bullying sub-scales, these two new sub-scales may be useful in developing profiles of children, particularly children who have preferences for either physical activity or screen-viewing behaviors.

The analysis presented in this paper has focused on the reliability of the new scales and not the "validity" of the scales [52]. However, as the development of the scales and items were informed by extensive qualitative work and the items assess the issues raised in the qualitative work the items can be considered to have "face and content validity" [52]. The items included in the scales address new constructs that were identified in the qualitative work and which have not been reported before and therefore there is no existing scale against which to compare these items. As such we are unable to assess the "criterion validity" but in order to provide an indication of the potential utility of these scales we have provided information on the associations with physical activity the key behavior to which they are hypothesized to relate.

### Strengths/limitations

This study has developed and provided reliability information on new scales that provide information on how friends and peers influence children's physical activity patterns. However, while we have been able to demonstrate the reliability of these measures, the relatively small sample limits our ability to examine associations with physical activity and particularly limits our ability to examine sex-specific associations. Moreover, as we did not collect family structure data we are unable to examine if responses for the new *Parental Influence scale *which assesses the influence of weekday and weekend parents differed by the time that children spent with different parents. It is also important to recognize that the development and piloting of this questionnaire has been conducted in one area of the United Kingdom, and as such the concepts assessed could be influenced by the location in which the children reside and may require refinement for use in other countries. Moreover, the development and factor analysis has only been conducted in a single sample and, as such, confirmation of the factor structure in another sample may be required before widespread adoption.

## Conclusion

Three scales that assess how parents, friends and group normative values may be associated with children's physical activity have been shown to be reliable and internally consistent. Initial analyses suggests that these measures will provide new information on the factors that influence children's physical activity but more research in a larger dataset is required to identify how associations may differ by sex and participant characteristics. They also provide further indication of the importance of conceptualizing physical activity as social-context specific with different parental and peer influences in action in different settings and time periods in the week.

## Competing interests

The authors declare that they have no competing interests.

## Authors' contributions

This paper was conceived and drafted by RJ. All authors provided critical input into the design of the research, drafting of the paper and provided edits to the paper. All authors read and approved the final manuscript.

## Supplementary Material

Additional file 1 **Table S1: Correlations among newly derived factors**. Correlations among the factors derived from the new physical activity questionnaire scales Click here for file

Additional file 2 **Table S2: Correlations between newly derived factors derived from the three scales and accelerometer assessed physical activity**. Correlations among the factors derived from the new physical activity questionnaire scales and physical activity stratified by gender Click here for file
