# Supplementary material for: Development of scales to assess children's perceptions of friend and parental influences on physical activity
Source: Int J Behav Nutr Phys Act. 2009 Oct 12;6:67. doi: 10.1186/1479-5868-6-67 (PMC2763850; doi:10.1186/1479-5868-6-67)
Supplement: Additional file 1 — Table S1: Correlations among newly derived factors. Correlations among the factors derived from the new physical activity questionnaire scales [file 1479-5868-6-67-S1.DOC]

**Table S1**: Correlations among newly derived factors

|  | **1** | **2** | **3** | **4** | **5** | **6** | **7** | **8** | **9** | **10** |
| --- | --- | --- | --- | --- | --- | --- | --- | --- | --- | --- |
| **General parenting support - 1** |  |  |  |  |  |  |  |  |  |  |
| **Active parent - 2** | .262** |  |  |  |  |  |  |  |  |  |
| **Parental past activity - 3** | .040 | -.091 |  |  |  |  |  |  |  |  |
| **Guiding support -4** | .046 | .119 | .238** |  |  |  |  |  |  |  |
| **Avoid bullying - 5** | -.026 | .024 | .027 | .229** |  |  |  |  |  |  |
| **Social sedentary - 6** | -.123 | -.010 | .160* | .216** | .426** |  |  |  |  |  |
| **Social affiliation - 7** | -.027 | .095 | .049 | .264** | .521** | .401** |  |  |  |  |
| **Neighborhood friends - 8** | .015 | .126 | .100 | .127 | .447** | .390** | .328** |  |  |  |
| **Sedentary norms - 9** | .117 | .108 | .097 | .045 | .168* | .139 | .176** | .043 |  |  |
| **Active norms - 10** | .340** | .243** | .034 | -.043 | ..014 | -.019 | .-.047 | .-.040 | .102 |  |
| **Teasing norms - 11** | -.182* | .035 | .147 | .019 | .427** | .174* | .255** | .133 | .169* | -.086 |

*P<.05 **P<.01
