# Supplementary material for: Development of scales to assess children's perceptions of friend and parental influences on physical activity
Source: Int J Behav Nutr Phys Act. 2009 Oct 12;6:67. doi: 10.1186/1479-5868-6-67 (PMC2763850; doi:10.1186/1479-5868-6-67)
Supplement: Additional file 2 — Table S2: Correlations between newly derived factors derived from the three scales and accelerometer assessed physical activity. Correlations among the factors derived from the new physical activity questionnaire scales and physical activity stratified by gender [file 1479-5868-6-67-S2.DOC]

**Table S2**: Correlations between newly derived factors derived from the three scales and accelerometer assessed physical activity

|  | **Girls n = 67** | | | | **Boys n = 64** | | | |
| --- | --- | --- | --- | --- | --- | --- | --- | --- |
|  | **Sed Mins** | **Light Mins** | **MVPA Mins** | **Counts per min** | **Sed Mins** | **Light Mins** | **MVPA Mins** | **Counts per min** |
| **General parenting support** | -.171 | .135 | .044 | .098 | -.027 | -.024 | .086 | .068 |
| **Active parent** | -.226 | .165 | .024 | .084 | -.062 | .178 | .123 | .160 |
| **Parental past activity** | .136 | -.307* | .077 | -.175 | -.074 | .057 | .123 | .033 |
| **Guiding support** | -.049 | .005 | -.042 | -.101 | .040 | -.077 | -.004 | -.212 |
| **Avoid bullying** | .197 | -.137 | -.255* | -.268* | -.110 | .098 | .234 | .126 |
| **Social sedentary** | .273* | -.173 | -.126 | -.249* | -.018 | -.061 | .187 | .061 |
| **Social affiliation** | .016 | .116 | -.113 | -.020 | -.196 | -.010 | .253* | .102 |
| **Neighborhood friends** | .090 | -.063 | -.044 | -.099 | -.041 | -.003 | .212 | .145 |
| **Sedentary norms** | -.017 | -.025 | .045 | .034 | -.094 | .013 | .242 | .160 |
| **Teasing norms** | .220 | -.170 | -.160 | -.232 | -.167 | .168 | .195 | .158 |
| **Active norms** | -.142 | -.002 | .215 | .141 | .015 | .140. | .024 | .102 |

*P<.05 **P<.01
